# Functional food awareness and perceptions in relation to information sources in older adults

**DOI:** 10.1186/1475-2891-13-44

**Published:** 2014-05-17

**Authors:** Meagan N Vella, Laura M Stratton, Judy Sheeshka, Alison M Duncan

**Affiliations:** 1 Department of Human Health and Nutritional Sciences, University of Guelph, 50 Stone Road East, Guelph, Ontario, Canada N1G 2W1; 2 Food, Nutrition and Dietetics, Victoria University, PO Box 14428, Melbourne, Victoria 8001, Australia

**Keywords:** Functional food, Older adult, Questionnaire, Nutrition information, Health claim

## Abstract

**Background:**

The functional food industry has experienced innovative and economic expansion, yet research into consumer perceptions of functional foods and their associated health claims is limited. Among consumers, older adults could benefit from functional foods due to age-related issues pertaining to food and health. The purpose of this research was to identify the need for information related to functional foods among older adults (≥60 years old) and to assess awareness and perceptions of health claims on functional food packages.

**Methods:**

Community-dwelling older adults (n = 200) completed a researcher administered questionnaire designed to collect information about functional foods including current consumption, motivating factors for consumption, perceived need for information, sources of information for functional foods and awareness of health claims.

**Results:**

Prevalence of functional food consumption among participants was 93.0%. Increased awareness and knowledge was the most commonly reported factor that would promote functional food consumption (85.5%) and 63.5% of participants wanted more information about functional foods with preferred sources being newspapers/magazines/books (68.5%) and food labels (66.1%). Participants were predominately (93.5%) aware of health claims on functional foods and those with more education were more likely to report being aware of health claims (p = 0.045).

**Conclusions:**

Although functional food consumption among older adults in this sample is high, there is a need for further information regarding functional foods. These results inform stakeholders regarding the potential for information to influence functional food acceptance among older adult consumers.

## Background

The functional food industry has experienced rapid growth and development in the Canadian marketplace [1]. The expansion of the functional food industry can be attributed to numerous factors including innovations in food science and technology, an aging population with growing health concerns, an evolving regulatory environment allowing health claims on foods and increased marketing of functional food products [2-5]. Fundamental to the advancement of functional foods has been the evolution of scientific and consumer interest in the ability of nutrition to prevent chronic disease and optimize health, which goes beyond the traditional focus on prevention of nutrient deficiency diseases [2,3,6,7]. Functional foods exemplify this evolution in food and health as they have been demonstrated to have physiological benefits and/or reduce the risk of chronic disease beyond basic nutritional functions [8]. However, the long-term marketplace success of functional foods is dependent on consumer acceptance, and attitudes and perceptions related to these products [9-11]. Related to this, the effective communication of nutrition and health information has been identified as a factor that can influence consumer acceptance of functional foods [12], yet further research is needed to elucidate consumer perceptions of nutrition and health information.

Among consumers, the awareness and perceptions of older adults in relation to functional foods is of particular interest, as this population could greatly benefit from the incorporation of functional foods into their diets [7]. The older adult population segment is rapidly increasing, with projections of those >65 years old in Canada increasing from 4.2 to 9.8 million between 2005 and 2036 and comprising up to 25% of the Canadian population by 2041 [13,14]. In parallel, chronic age-related diseases such as cardiovascular disease, cancer, osteoporosis and age-related macular degeneration are also increasing [3,15], posing a significant burden on the health care system [2]. Functional foods, with their bioactive constituents, are a potential strategy to mitigate the increased risk of chronic disease among older adults [2]. Since the older adult population interacts more frequently with health care providers [14], there is a need for an understanding of older adults’ awareness and perceptions of functional foods to inform proper advice in regards to functional foods and health.

Nutrition and health information, and the source of this information, has the potential to influence acceptance of functional food products by communicating the health benefits of such products [16]. Consumer research has suggested that consumers are more likely to substitute conventional foods for functional foods if they perceive the functional food products to be healthier [16-18]. However, unlike sensory characteristics of a food product, the health benefits of functional foods cannot be directly perceived by consumers, hence information pertaining to health benefits and the ways in which this information is communicated can influence perceptions of functional food products [16,17,19]. Consumer confidence and trust in the source of nutrition information are also core factors that influence acceptance of functional foods [16,18,20]. Health professionals, including physicians and dietitians, have been identified in consumer research as perceived credible sources of information pertaining to nutrition and health [21-25]. Family members and friends have also been identified by consumers to be trusted sources of nutrition and health information [21-23,25]. As nutrition and health information can influence consumer acceptance of functional food products, it is of value to establish the preferred sources of information and perceived need for more information regarding functional foods among older adult consumers.

Nutrition information sources on food labels, particularly health claims, are a valuable consumer educational tool that could influence acceptance of functional foods [12,18]. Consumer studies have demonstrated that the presence of health claims on functional food labels results in more favourable attitudes towards functional foods [16,26-28] and has a positive influence on consumers’ perceived healthiness of functional foods [29-31]. In contrast, other research has identified consumer skepticism with regards to the legitimacy of health claims on functional foods [21,23,25,32], the scientific evidence substantiating the health claim [21,23] and the legislation regulating the health claim [21,25]. In addition to investigating consumer attitudes towards health claims on functional foods, studies have explored the type of health claim preferred by consumers. Research has shown that health claims relating the food product to a physiological benefit or the reduction of disease risk tend to be preferred by consumers over claims relating to a psychological benefit, such as reduced tiredness or stress [20,33,34]. Nutrient function claims have also been found to be more convincing and attractive than nutrient content claims among Belgian consumers and both were favoured over disease risk reduction claims [35]. There is also evidence to suggest that nutrient content claims stating that the product contains a familiar component are perceived by consumers to be as equally advantageous as nutrient function or disease risk reduction claims as consumers are already aware of the health benefits of the component [16]. Ultimately, more research is needed to determine the type of health claim preferred by various consumer segments in order to inform food manufacturers and allow for targeted information provision [10-12,33].

While there is evidence that nutrition and health information has the potential to influence acceptance of functional foods, the awareness and understanding of this information among older adults, a key beneficiary of functional foods, has yet to be explored. There is substantial heterogeneity among consumers in terms of their needs, interests and perceptions related to nutrition and health information [12,36,37], yet the current research regarding consumer perceptions has not been sufficiently targeted. The purpose of the current study was to generate information related to functional food consumption among a sample of older adults through an exploration of the sources of information related to functional foods and the awareness, perceptions and understanding of health claims on functional food products.

## Methods

### Participant recruitment and screening

A total of 200 community-dwelling older adults were recruited through study flyers, advertisements in local newspapers and senior community centre newsletters, blog postings on websites targeted towards older adults, social media, emails circulated to University of Guelph campus departments and tables set up in senior community centres and malls. The study was advertised as a food survey to minimize bias in participant recruitment with respect to functional food awareness and consumption. Individuals were included in the study if they were ≥60 years of age and were living independently within the community. Exclusionary criteria included use of any meal-assisted services or any indication of cognitive dysfunction that would interfere with completion of the study questionnaire. Participant eligibility was determined over the phone or via email utilizing an eligibility questionnaire. All participants provided written informed consent and the research protocol was approved by the University of Guelph Human Research Ethics Board (REB#10SE012).

### Data collection

Participants attended study appointments at the Human Nutraceutical Research Unit (HNRU) at the University of Guelph. The study appointment included review and completion of the study consent form followed by completion of a questionnaire designed to explore functional food consumption. Upon completion of the study questionnaire, participants received a Dietitians of Canada cookbook.

The study questionnaire was a part of a larger, comprehensive questionnaire designed to explore numerous factors related to functional food consumption among older adults. A combination of open- and close-ended questions was utilized to generate quantitative and qualitative data regarding sources of information related to functional foods and awareness, perceptions and understanding of health claims on functional food products. These questions explored current consumption of functional foods, motivating factors that would increase functional food consumption, current sources of information for functional foods, need for more information regarding functional foods and the preferred sources of that information. Health claims on functional foods were explored in terms of awareness, perceptions and understanding of nutrient content claims, nutrient function claims and disease risk reduction claims. The questionnaire also collected information on medical and demographic characteristics.

To ensure that the participant understood and answered each question fully, the study questionnaire was administered by a researcher using an interactive process. Throughout this process, to increase the participant’s awareness and understanding of key concepts related to functional foods, information sheets were presented to describe and establish the definition of functional foods, nutrient content claims, nutrient function claims and disease risk reduction claims. A functional food was defined in partial accordance with the Health Canada definition of a food that is “similar in appearance to, or may be, a conventional food, is consumed as a part of a usual diet and is demonstrated to have physiological benefits and/or reduce the risk of chronic disease beyond basic nutritional functions, i.e. they contain a bioactive compound” [8]. However, the current study excluded conventional foods to limit the definition of a functional food to foods that had undergone processing or manipulation to add or increase the level of a bioactive. Nutrient content claims were described to participants using an information sheet as claims on food packaging that indicate the presence of a specific nutrient, but do not explicitly relate the nutrient to health. Nutrient function claims were described to participants as health claims on food packaging that link a component of the product to the maintenance of a physiological function or to physical or mental performance, without explicit reference to a disease. Disease risk reduction claims were described to participants as health claims on food packaging that link a component of the product to a reduced risk of a diet-related disease or condition.

### Data and statistical analysis

All data from the study questionnaire were entered into excel spreadsheets. Quantitative data was analyzed through the calculation of summary statistics including frequencies and percentages. Chi-square analysis was used to consider gender, education and income for quantitative variables with a yes/no response related to information about functional foods and awareness and perceptions of health claims. Consideration of gender, education and income is consistent with previous literature which has investigated these factors as core socio-demographic variables that have the potential to influence functional food perceptions [10,36,38,39]. Qualitative data was examined for common responses, which were then organized into groupings, tallied and summarized as frequencies and percentages. All statistical analyses were performed using the Statistical Analysis System (version 9.3, Cary, NC, USA) with p < 0.05 considered statistically significant.

## Results

### Participant characteristics

A total of 200 community-dwelling older adults (70.8 ± 7.17 years old) completed the study questionnaire and are characterized in Table 1. The prevalence of functional food consumption among participants was 93.0%.

**Table 1:** **Participant demographic and lifestyle characteristics (n = 200)**^**a,b**^

	**n**	**%**
Gender		
Male	60	30.0
Female	140	70.0
Age (years)		
60-64	52	26.0
65-69	42	21.0
70-74	43	21.5
75-79	37	18.5
80 +	26	13.0
Ethnicity		
Caucasian	190	95.0
African	1	0.50
Asian	5	2.50
Other	4	2.00
Marital status		
Single	17	8.50
Married/Common-law	117	58.5
Divorced	31	15.5
Widowed	35	17.5
Education		
Some high school	8	4.00
High school graduate	20	10.0
Some college or university	38	19.0
College or university graduate	84	42.0
Post-graduate degree	50	25.0
Employment Status		
Currently employed	35	17.5
Not currently employed	165	82.5
Retired		
Yes	164	98.8
No	2	1.20
Annual household income (Canadian $)		
≤ $24,999	27	15.3
$25,000-$49,999	42	23.9
$50,000-$74,999	50	28.4
$75,000-$99,999	28	15.9
≥ $100,000	29	16.5
Frequency of visits to a physician		
Once a month	12	6.00
Every 3–4 months	48	24.0
Every 6 months	50	25.0
Once a year	72	36.0
< Once a year	18	9.00
Frequency of visits to a registered dietitian		
Once a month	3	1.50
Every 3–4 months	5	2.50
Every 6 months	1	0.50
Once a year	8	4.00
< Once a year	38	19.0
Never	145	72.5
Number of prescription medications used		
0	42	21.0
1	37	18.5
2	40	20.0
3	23	11.5
4 +	58	29.0
Number of non-prescription medications used		
0	17	8.50
1	20	10.0
2	32	16.0
3	38	19.0
4 +	93	46.5

^a^All data is participant-reported.

^b^Sample sizes may total less than 200 as participants had the option of not answering a question.

### Knowledge and information about functional foods

**Figure 1:**
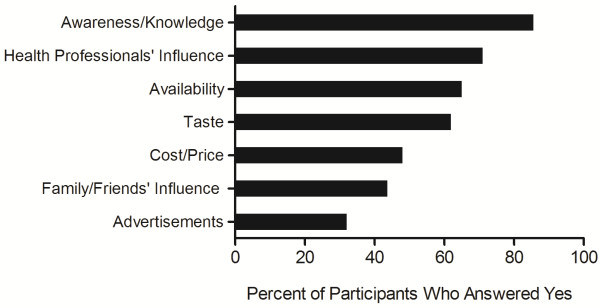
**Factors reported to increase functional food consumption.** Data is expressed as the percent of participants (n = 200) that answered “yes” when asked if the factor would increase their consumption of functional foods.

An increased awareness and knowledge (85.5% of participants) and the influence of a health professional (71.0%) were the most frequently identified factors that would promote functional food consumption among participants (Figure 1). Cost/price ranked fifth as a factor that would promote consumption (48.0%) and participants with an annual household income ≥ $50,000 were less likely to report that cost of functional foods was a factor compared to those with an annual household income < $50,000 (p = 0.002). There were no other significant differences among gender, education or income groups for any factors that would promote functional food consumption.

**Figure 2:**
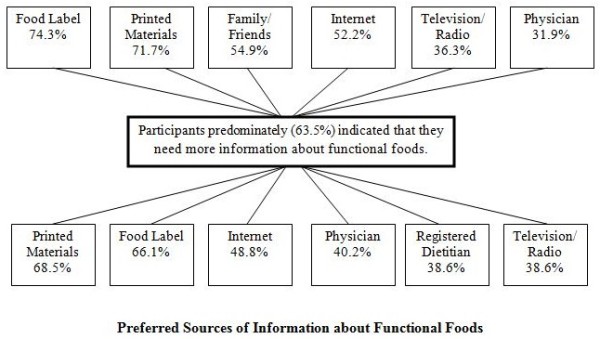
**Current and preferred sources of information about functional foods.** Data is expressed as the percent of participants (n = 200) who selected the identified source of information from a list. Participants were able to select more than one source of information. “Printed materials” refers to newspapers, magazines and/or books.

Related to awareness and knowledge, over half of participants (56.5%) reported that they actively seek out information about functional foods with the most common sources of information being food labels (74.3%), newspapers, magazines and/or books (71.7%) and family and/or friends (54.9%) (Figure 2). Among the 63.5% of participants who indicated they needed further information about functional foods, the preferred sources included newspapers, magazines and/or books (68.5%), food labels (66.1%) and the internet (48.8%) (Figure 2) and the type of information needed was most frequently identified as that pertaining to the health benefits of functional foods (26.8%), the bioactive ingredients associated with functional foods (15.8%) and the risks/adverse effects related to functional foods (14.2%). There were no significant differences among gender, education or income groups for any variables related to information about functional foods.

### Health claims as they relate to functional foods

The majority of participants (93.5%) reported that they were aware of the health claims that are present on some food labels and of those that were aware, 91.0% indicated that they read the health claims. Participants with a higher education level (college, university or post-graduate degree) were more likely to report being aware of the health claims compared to those with a lower education level (some college or university, high school graduate or some high school) (p = 0.045), with no significant differences among gender or income groups.

**Figure 3:**
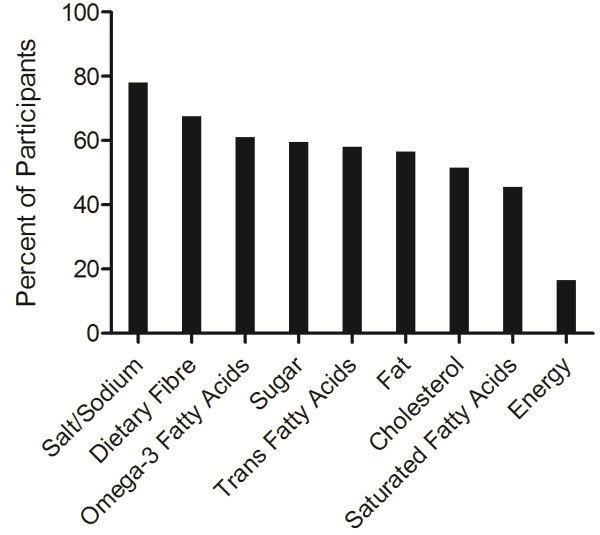
**Nutrients mentioned in a nutrient content claim that would increase participants’ functional food consumption.** Participants (n = 200) were able to select more than one nutrient. Nutrient content claims were described to participants using an information sheet as claims on food packaging that indicate the presence of a specific nutrient but do not explicitly relate the nutrient to health.

Nutrient content claims were predominately reported to be informative (80.8% of participants) and the majority of participants (68.5%) indicated that the presence of a nutrient content claim on a functional food label would increase the likelihood that they would consume that functional food product, with no significant differences found in gender, education or income groups. The most frequently reported nutrients that, when mentioned in some type of nutrient content claim on a functional food product, would increase participants’ consumption of the product included salt/sodium (78.0%), dietary fibre (67.5%) and omega-3 fatty acids (61.0%) (Figure 3).

Nutrient function claims were predominately reported to be informative (76.7%) and the majority of participants (63.5%) indicated that the presence of a nutrient function claim on a functional food label would increase the likelihood that they would consume that functional food product, with no significant differences found in gender, education or income groups. The primary functions or biological roles that, when mentioned in a nutrient function claim on a functional food product, would increase participants’ consumption of the product included “helps lower cholesterol” (60.5%), “factor in the formation and maintenance of bones and teeth” (56.5%) and “dietary antioxidant” (55.5%) (Table 2).

**Table 2:** **Functions or biological roles mentioned in nutrient function claims that would increase functional food consumption**^**a,b**^

**Function or biological role**	**Percent of participants (n = 200)**
Helps lower cholesterol	60.5
Factor in the formation and maintenance of bones and teeth	56.5
Dietary antioxidant	55.5
Factor in the maintenance of good health	44.5
Helps build antibodies	44.5
Promotes regularity	42.5
Helps build and repair body tissues	40.0
Aids in maintaining the health of skin and membranes	34.0
Aids in red blood cell formation	34.0

^a^Participants were able to select more than one function or biological role.

^b^Nutrient function claims were described to participants using an information sheet as health claims on food packaging that link a component of the product to the maintenance of a physiological function or to physical or mental performance, without explicit reference to a disease.

**Figure 4:**
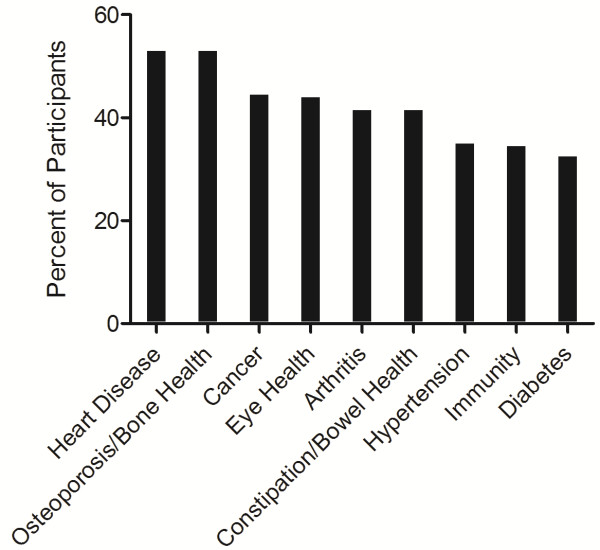
**Health areas mentioned in a disease risk reduction claim that would increase participants’ functional food consumption.** Participants (n = 200) were able to select more than one health area. Disease risk reduction claims were described to participants using an information sheet as health claims on food packaging that link a component of the product to a reduced risk of a diet-related disease or condition.

Disease risk reduction claims were predominately reported to be informative (67.3%). While 53.8% of participants indicated that the presence of a disease risk reduction claim on a functional food label would increase the likelihood that they would consume that functional food product, gender was a significant factor (females more frequently responded yes (p = 0.03)), with no significant differences among education or income groups. The primary health areas that, when mentioned in a disease risk reduction claim on a functional food product, would increase participants’ consumption of the product included heart disease (53.0%), osteoporosis/bone health (53.0%) and cancer (44.5%) (Figure 4).

In regards to overall understanding of the claims, the majority of participants did not find nutrient content (63.5%), nutrient function (66.0%), or disease risk reduction (74.8%) claims to be confusing. For disease risk reduction claims, education significantly influenced this result in that those with a college, university or post-graduate degree more frequently reported that they did not find them confusing compared to participants with a lower level of education (some college or university, high school graduate or some high school) (p = 0.02). Of those participants who provided a reason for their confusion regarding the claims, the primary reason specified for nutrient content claims was that they did not contain enough information (37.1%), with 21.6% of participants stating that they would further investigate the rest of the label, including the Nutrition Facts table. For both nutrient function and disease risk reduction claims, the primary reason for confusion specified was that participants were skeptical and/or had a lack of trust in the claims (39.5% and 50.6%, respectively). When asked to compare nutrient content claims, nutrient function claims and disease risk reduction claims, participants primarily reported that the claims were equally informative (34.5%) or that nutrient content claims were most informative (34.5%).

## Discussion

The aim of the current study was to generate information related to functional food consumption among a sample of 200 older adults using a researcher-administered questionnaire. To date, there has been minimal investigation into the perception of information related to functional foods among older adults, a consumer segment that is poised to benefit from the incorporation of functional foods in their diets due to age-related health issues [7]. Hence, the current study advances this area of knowledge to provide information that can be utilized by stakeholders including health care professionals and the food industry to improve functional food acceptance among older adults.

### Participant characteristics

Reflective of the rapidly aging population in Canada [13,14], all participants in the current study were 60 years of age or older (mean age of 70.8 ± 7.17 years old). Previous investigations into consumer attitudes, knowledge and perceptions of functional foods and their associated health claims have not focused on older adults and rather explored a broader age demographic [16,31,33,40-43] and have primarily been geographically limited to Europe [16,24,31,33,40-42] and Australia [21,25,43]. Participants in the current study were predominately female (70.0%) and Caucasian (95.0%) which reflects the higher proportion of women and those of Caucasian ethnicity within the Canadian older adult population [14]. However, participants had a higher level of education and a greater annual household income relative to the overall Canadian population of older adults [14], which limits generalizability of the study results.

### Knowledge and information about functional foods

An increased awareness and knowledge about functional foods was the most frequently identified factor (85.5% of participants) that would increase functional food consumption. This is consistent with results of a Portuguese study in which knowledge and familiarity with the functional food product were among the top factors influencing choices of functional foods [44]. Other questionnaire studies have also shown that knowledge, particularly with respect to the nutritional attributes and potential health benefits of functional foods, can increase consumers’ perceived healthiness of functional foods [19] and willingness to consume functional foods [19,45]. Participants in the current study also frequently reported (71.0% of participants) that the influence of a health professional would increase their consumption of functional foods, suggesting that the advice of health professionals has the potential to influence functional food consumption among older adults. This is in line with previous research that has shown that consumers perceive health professionals to be credible and trustworthy sources of information regarding functional foods [21,23-25,46].

Related to information, the majority (56.5%) of participants in the current study indicated that they actively seek out information about functional foods. The most common sources of information about functional foods utilized by participants were food labels (74.3%), newspapers, magazines and/or books (71.7%) and family and/or friends (54.9%). These results are comparable to those of a Swedish questionnaire study in which the most common sources of information for functional foods were identified as television, advertisements in newspapers or magazines and food packaging. Furthermore, a 2008 Canadian population study investigating nutrition knowledge, attitudes and behaviours found that Canadian consumers obtain food and nutrition information from easily accessible sources and that the most common sources utilized were food labels, the internet and magazines, newspapers and books [47]. Also consistent with the current study, focus group research has identified family and/or friends as key sources of information pertaining to functional foods [22,23,25]. While some studies have suggested that consumers distrust the information on food labels [22,25], the results of the current study demonstrate that the older adult consumers primarily utilize food labels as a source of information about functional foods. Noteworthy is that although participants in the current study predominately reported that the influence of a health professional would increase their functional food consumption, health professionals were not among the common sources of information for functional foods. Hence, there is great potential for health professionals to promote increased functional food acceptance and consumption among older adults by serving as a source of credible information pertaining to functional foods.

Participants in the current study predominately (63.5%) indicated that they need more information about functional foods and would prefer to receive this information from newspapers, magazines and/or books (68.5%), food labels (66.1%) and the internet (48.8%). As increased awareness and knowledge was the primary factor that would increase functional food consumption among this sample of older adults, functional food information coming from these sources is likely to influence awareness and consumption. The type of information participants would like to receive about functional foods largely pertained to the health benefits of functional foods (26.8%), the bioactive ingredients in functional foods (15.8%) and the risks/adverse effects related to functional foods (14.2%). Similarly, participants of a focus group study conducted in Sweden expressed a lack of knowledge regarding which ingredients in functional foods have an effect on the body and how these ingredients impact health [24]. Previous questionnaire studies conducted in Uruguayan [19] and American [45] adults have also suggested that the most influential information with respect to consumer acceptance of functional foods is that which communicates the relationship between the functional ingredient and health. While a relatively low percentage (14.2%) of participants in the current study perceived the need for information pertaining to the risk of adverse/negative effects of functional foods, previous focus group studies have highlighted consumer concerns regarding the safety of functional foods, particularly with regards to the potential long-term effects on health [48], the possibility of overconsumption of bioactive ingredients [24,25] and negative interactions between functional foods or between ingredients within a functional food product [24,48]. Overall, it is evident that older adults in this sample are motivated to seek out information about functional foods and more information, particularly with respect to the health benefits of functional foods, has the potential to increase functional food consumption among this consumer segment.

### Health claims as they relate to functional foods

The majority of participants (93.5%) in the current study reported that they were aware of the health claims on food labels. Of those that were aware, 91.0% indicated that they read the health claims which is relatively higher than the prevalence of food label use identified in studies conducted in France [49], Uruguay [11], New Zealand [50] and the United Kingdom [41] which ranged from 27.0% to 82.0% of participants. This difference may be due to the fact that participants in the current study were asked specifically about their awareness and use of health claims on food labels, while the mentioned studies inquired about overall food label use among participants [11,41,49,50]. It should also be noted that these investigations were conducted in countries with varying regulations regarding food labels and health claims, thus limiting comparisons to the current study. The relatively high prevalence of health claim use in the current study may also be due to over-reporting by participants; however, some studies have shown that older consumers tend to be more health-oriented and have a greater interest in healthy eating [41,51] which may translate into greater use and interest in nutrition information [37,52]. In the current study, participants with a higher education level were significantly more likely to report being aware of health claims compared to those with a lower education level, which is comparable to previous research that has demonstrated a greater use of nutrition labels among individuals with a higher level of education [37,53]. Overall, these findings indicate that older adult consumers in this sample are cognisant of the health claims on functional food products and that level of education plays a role in consumer awareness of health claims.

The majority of older adult participants in the current study indicated that they found nutrient content claims, nutrient function claims, and disease risk reduction claims to be informative and that the presence of these health claims on a functional food label would increase their likelihood of consuming that functional food product. Similarly, results of a number of questionnaire [27,31,54], interview-based [55] and purchase simulation [40] studies have shown that the presence of a health claim on a functional food product has a positive effect on purchase intention and the likelihood of consumption of the product. The presence of health claims on functional food products has been shown to influence consumers’ perceptions of the healthiness and nutritional value of the products [26,30,31,54,55], which may rationalize consumers’ reported preference for products with health claims. In the present study, females were significantly more likely than males to report that the presence of a disease risk reduction claim on a functional food label would increase the likelihood that they would consume that product. Although a number of previous studies conducted in Belgian [10,31,35] and Finnish [56] adults have found weak associations between socio-demographic variables and attitudes towards functional foods and their health claims, some studies have demonstrated that women are more interested in nutrition information [52] and healthy eating [41,57] than men. Ultimately, further research is needed in order to elucidate the relationship between socio-demographic variables and consumer perceptions of health claims on functional food products in order to develop targeted nutrition information that will appeal to specific consumer groups.

Participants in the current study were asked to indicate which nutrients, when mentioned in a nutrient content claim on a functional food product, would increase their consumption of the product. The most common responses were salt/sodium (78.0% of participants), dietary fibre (67.5%) and omega-3 fatty acids (61.0%) although responses were high for most of the nutrients listed, suggesting that older adults in this sample consider a number of food components when using nutrient content claims to make choices regarding functional food products. The frequent choice of a salt/sodium nutrient content claim in the current study is comparable to previous studies conducted in Australian [21] and English [41] adults in which participants most frequently searched for information on food labels regarding the content of fat, sugar, salt and calories. This finding may also be attributable to the current focus on reducing the sodium content in food products in Canada and the increased public awareness of health issues related to high sodium intake [58]. Dietary fibre and omega-3 fatty acids were also frequently chosen by participants in the current study as nutrients that, when mentioned in a nutrient content claim on a functional food label, would increase consumption, suggesting that the content of these bioactive ingredients is of particular interest to older adults when choosing functional food products. This information is of value from a food industry perspective as further development of functional food products which are low in sodium and contain these bioactives may be well accepted among older adult consumers.

When participants in the current study were asked to select the primary functions or biological roles that, when mentioned in a nutrient function claim on a functional food product, would increase the likelihood that they would consume the product, their most common responses were “helps lower cholesterol” (60.5%) and “factor in the formation and maintenance of bones and teeth” (56.5%). These findings are similar to the results of questionnaire studies conducted in a broader consumer demographic in the Netherlands [33], Trinidad [59] and Uruguay [11] in which claims for functional foods related to lowering or maintaining healthy cholesterol levels were among the top preferred claims, although claims related to strengthening the immune system and providing the body with energy were also among the top preferred claims [11,33,59]. In the current study, participants reported heart disease (53.0%), osteoporosis/bone health (53.0%) and cancer (44.5%) as the top health areas that, when mentioned in a disease risk reduction claim on a functional food product, would increase their consumption of the product. Claims on functional foods pertaining to a reduced risk of cancer and heart disease were also among the top preferred claims reported by participants in previous questionnaire studies [11,33] and it has been suggested that claims on functional food products relating to health areas that are of personal relevance to consumers are expected to be most successful in the marketplace [16,33,46]. The results of the current study suggest that the primary health concerns of older adults in this sample are heart health, maintenance of healthy cholesterol levels and osteoporosis/bone health. This information has the potential to drive the development of functional food products that bear claims relating to these health concerns, as they may be successful among older adult consumers.

With respect to understanding of health claims on functional food products, the majority of participants in this study did not find nutrient content (63.5%), nutrient function (66.0%), or disease risk reduction (74.8%) claims to be confusing. Furthermore, participants with a higher level of education were significantly more likely to report that they did not find disease risk reduction claims confusing compared to those with a lower level of education, suggesting that education not only contributes to awareness of health claims among older adults in this sample, but also to understanding of the claims. However, as highlighted by a large-scale cross-national study conducted in Italy, Germany, the United Kingdom and the United Sates, numerous factors are likely to influence consumer understanding of health claims including knowledge pertaining to the claim or the food component mentioned in the claim, familiarity with the product and familiarity with the terminology used in the claim [60]. Among participants in the current study, the main reason for confusion regarding nutrient content claims was that they did not contain enough information (37.1%), with 21.6% of participants stating that they would look to the rest of the label, including the nutrition facts table, for more information. This is contrary to an interview-based study conducted by the United States Food and Drug Administration which found that in the presence of a nutrient-health claim, consumers tend to truncate their information search to only the claim itself and do not typically investigate the nutrition information on the rest of the label [55]. However, coinciding with the frequent use of the food label as a source of information for functional foods, it is evident that older adults in the present study utilize the nutrition information present on food labels to increase their knowledge and understanding of the health claims associated with functional food products.

Of the participants in the present study who found nutrient function and disease risk reduction claims to be confusing, the primary reason for confusion specified was that participants were skeptical and/or had a lack of trust in the claims (39.5% and 50.6%, respectively). A number of focus group studies examining consumer beliefs and attitudes towards functional foods and their associated health claims also found that participants exhibited skepticism towards health claims, which was related to a lack of trust in the information provided by food manufacturers [21,22,24,25]. Participants sampled from Canadian [20], Australian [25] and Uruguayan [19] adults have communicated that they would be more trusting of claims that were substantiated by scientific evidence and verified by the government. These data highlight the importance of consumer trust in the information pertaining to functional foods, which is a key factor for functional food acceptance [18,34]. While the minority of participants in the current study expressed skepticism towards the health claims on functional foods, the implementation of educational campaigns designed to inform consumers of the thorough regulatory process required to approve health claims for food products in Canada may improve consumers’ perceptions of the credibility of health claims.

In order to elucidate the type of health claim preferred by older adults in the present study, participants were asked whether they found nutrient content claims, nutrient function claims, or disease risk reduction claims to be most informative. Participants primarily reported that the claims were equally informative (34.5%) or that nutrient content claims were most informative (34.5%). Numerous previous studies have attempted to determine the type of health claim preferred by consumers [16,20,33,35,42], however the results have been inconsistent which may be related to differences in the consumer demographics examined. The preferred type of health claim for functional foods will differ depending on the consumer group examined and may change over time as consumers develop more knowledge pertaining to functional foods and health claims [42]. Despite these inconsistencies, the results of studies conducted in the United States [61] and Finland [16] demonstrated that participants prefer shorter claims relating to the content of nutritional components such as fat or probiotics, which coincides with the preference for nutrient content claims observed in the current study. Ultimately, these results indicate that older adult consumers perceive health claims on functional food products to be informative and demonstrate some preference toward claims that indicate the presence of specific nutrients or food components.

### Study limitations and strengths

While the current study thoroughly explored information sources for functional foods and the awareness and perceptions of health claims on functional food products among a sample of older adults, it is not without limitations. All data collected was self-reported and therefore there may be discrepancies between the reported information and participants’ actual understanding and perceptions related to functional foods and health claims. It is also limiting that participants were recruited from a specific geographic area and were predominately Caucasian and well-educated with a relatively high annual household income, all of which hinders generalizability of the results.

Despite these limitations, the current study had strength in that it utilized a researcher-administered questionnaire which allowed for researcher and participant interaction, ensuring understanding of the concepts examined and the completeness of the data. In addition, the study questionnaire consisted of a variety of open- and close-ended questions which enabled the collection of a wide breadth of data. This also allowed for the collection of quantitative data, yet participants were able to qualitatively describe their perceptions related to functional foods and health claims. To date, there has been limited investigation into the awareness and perceptions of health claims from a Canadian regulatory perspective and thus the current study greatly contributes to this area of knowledge. The present study also had broad inclusion criteria which allowed for the recruitment of a wide sample of older adults from which to gather data regarding functional foods, information sources and health claims.

## Conclusions

In summary, this study provides insight into the current and preferred sources of information for functional foods, as well as the awareness, perceptions and understanding of health claims on functional foods among the older adult consumer segment. Among participants, an increased awareness and knowledge pertaining to functional foods was the most frequently reported factor that would increase consumption and the majority of participants indicated that they actively seek out information about functional foods, suggesting that older adults are motivated to expand their knowledge and understanding of functional food products. The food label was also identified as a primary source of information pertaining to functional foods; however, older adults in the current study perceived the need for more information, specifically with regards to the health benefits of functional foods. Further information from the preferred sources of information identified by participants in the current study has the potential to improve functional food acceptance and consumption. With respect to health claims as a source of information for functional foods, participants in the current study predominately reported that they found nutrient content, nutrient function and disease risk reduction claims to be informative. The results also demonstrate that the presence of health claims on functional foods, specifically claims related to nutrients or health concerns that are of particular interest to older adults, can increase the likelihood of consumption of a functional food product. Overall, the results of the current study provide valuable information regarding the needs and perceptions of nutrition and health information on functional foods among the older adult consumer segment. Ultimately, this information can be utilized by numerous stakeholders including health professionals and the food industry to further educate older adults regarding the age-related health benefits of consuming functional foods.

## Competing interests

The authors declare that they have no competing interests.

## Authors’ contributions

AMD and JS designed the study and secured the funding. MNV and LMS co-coordinated the participant recruitment, data collection and data entry. MNV and AMD completed the statistical analysis and interpreted the results. MNV summarized the results, drafted the manuscript and advanced it to its final form. AMD directed the data collection, data entry and analysis, results interpretation and manuscript preparation and finalization. All authors read and approved the final manuscript.
